# What do Cochrane systematic reviews say about congenital vascular anomalies and hemangiomas? A narrative review

**DOI:** 10.1590/1516-3180.2021.0374.R2.15092021

**Published:** 2022-03-14

**Authors:** Henrique Jorge Guedes, Danielle Akemi Bergara Kuramoto, Rebeca Mangabeira Correia, Brena Costa Santos, Amanda da Cunha Borges, Mariana Raffo Pereda, Ana Laura e Silva Aidar, Francisco Amadeu Pereira, Fabio Cabral de Freitas Amaral, Vladimir Tonello de Vasconcelos, Wellington Gianoti Lustre, Jorge Eduardo de Amorim, Ronald Luiz Gomes Flumignan, Luis Carlos Uta Nakano, José Carlos Costa Baptista-Silva

**Affiliations:** I MD, PhD. Adjunct Professor, Division of Vascular and Endovascular Surgery, Universidade Federal de São Paulo (UNIFESP), São Paulo (SP), Brazil.; II MD. Resident, Division of Vascular and Endovascular Surgery, Universidade Federal de São Paulo (UNIFESP), São Paulo (SP), Brazil.; III MD. Resident, Division of Vascular and Endovascular Surgery, Universidade Federal de São Paulo (UNIFESP), São Paulo (SP), Brazil.; IV MD. Resident, Division of Vascular and Endovascular Surgery, Universidade Federal de São Paulo (UNIFESP), São Paulo (SP), Brazil.; V MD. Resident, Division of Vascular and Endovascular Surgery, Universidade Federal de São Paulo (UNIFESP), São Paulo (SP), Brazil.; VI MD. Resident, Division of Vascular and Endovascular Surgery, Universidade Federal de São Paulo (UNIFESP), São Paulo (SP), Brazil.; VII MD. Resident, Division of Vascular and Endovascular Surgery, Universidade Federal de São Paulo (UNIFESP), São Paulo (SP), Brazil.; VIII MD. Resident, Division of Vascular and Endovascular Surgery, Universidade Federal de São Paulo (UNIFESP), São Paulo (SP), Brazil.; IX MD. Postgraduate Student, Division of Vascular and Endovascular Surgery, Universidade Federal de São Paulo (UNIFESP), São Paulo (SP), Brazil.; X MD, PhD. Adjunct Professor, Division of Vascular and Endovascular Surgery, Universidade Federal de São Paulo (UNIFESP), São Paulo (SP), Brazil.; XI MD, MSc. Assistant Professor, Division of Vascular and Endovascular Surgery, Universidade Federal de São Paulo (UNIFESP), São Paulo (SP), Brazil.; XII MD, PhD. Adjunct Professor, Division of Vascular and Endovascular Surgery, Universidade Federal de São Paulo (UNIFESP), São Paulo (SP), Brazil.; XIII MD, PhD. Full Professor, Division of Vascular and Endovascular Surgery, Universidade Federal de São Paulo (UNIFESP), São Paulo (SP), Brazil.; XIV MD, PhD. Full Professor, Division of Vascular and Endovascular Surgery, Universidade Federal de São Paulo (UNIFESP), São Paulo (SP), Brazil.; XV MD, PhD. Full Professor, Division of Vascular and Endovascular Surgery, Universidade Federal de São Paulo (UNIFESP), São Paulo (SP), Brazil.

**Keywords:** Vascular malformations, Hemangioma, Arteriovenous malformations, Lasers dye, Laser therapy, Port-wine stain, Congenital vascular anomalies, Laser treatment

## Abstract

**BACKGROUND:**

Congenital vascular anomalies and hemangiomas (CVAH) such as infantile hemangiomas, port-wine stains and brain arteriovenous malformations (AVMs) impair patients’ lives and may require treatment if complications occur. However, a great variety of treatments for those conditions exist and the best interventions remain under discussion.

**OBJECTIVE:**

To summarize Cochrane systematic review (SR) evidence on treatments for CVAH.

**DESIGN AND SETTING:**

Review of SRs conducted in the Division of Vascular and Endovascular Surgery of Universidade Federal de São Paulo, Brazil.

**METHODS:**

A broad search was conducted on March 9, 2021, in the Cochrane Database of Systematic Reviews to retrieve any Cochrane SRs that assessed treatments for CVAH. The key characteristics and results of all SRs included were summarized and discussed.

**RESULTS:**

A total of three SRs fulfilled the inclusion criteria and were presented as a qualitative synthesis. One SR reported a significant clinical reduction of skin redness by at least 20%, with more pain, among 103 participants with port-wine stains. One SR reported that propranolol improved the likelihood of clearance 13 to 16-fold among 312 children with hemangiomas. One SR reported that the relative risk of death or dependence was 2.53 times greater in the intervention arm than with conservative management, among 218 participants with brain AVMs.

**CONCLUSION:**

Cochrane reviews suggest that treatment of port-wine stains with pulsed-dye laser improves redness; propranolol remains the best option for infantile hemangiomas; and conservative management seems to be superior to surgical intervention for treating brain AVMs.

## INTRODUCTION

Vascular anomalies are characterized by a disorder in blood vessels, either in structure or growth, and can affect arteries, veins and lymphatic vessels. These lesions are usually detected in children and account for 20%-30% of pediatric soft-tissue tumors. According to the International Society for the Study of Vascular Anomalies (ISSVA), vascular anomalies can be divided into tumors and vascular malformations.^1–3^

Vascular malformations are complex lesions that do not regress or disappear spontaneously and are subclassified as simple or combined. The combined lesions are further divided into ‘of major named vessels’ or ‘associated with other anomalies’.^1–4^ These condition affect 1.5% of the general population and have a wide variety of clinical presentations, such as disfiguration, coagulopathy (bleeding or thrombosis), organic or musculoskeletal dysfunction and pain, along with variation in the evolution of the clinical condition over time. Because of this heterogeneity, in terms of both origin and clinical status and evolution, there are several treatment options, requiring multidisciplinary follow-up to reduce the impact on quality of life.^5–7^

In contrast, vascular tumors are characterized by high rates of vascular cell proliferation. They are classified as benign, locally aggressive or borderline, or malignant.^2,8^ Hemangiomas are benign-subtype tumors than can be divided into infantile or congenital types.^2^ Despite the ISSVA nomenclature, both of these types of tumor are congenital. They have been described as proliferations of endothelial cells and growths of new vessels (angiogenesis) that usually flourish in the first weeks of life and tend to start to regress over time.^4,6,9^ Infantile hemangiomas are the most common soft-tissue tumor of infancy and occur in nearly 5% of the population.^10,11^ As hemangiomas tend to regress in childhood or during puberty, many tumors do not require treatment. However, infantile hemangiomas may need treatment when they follow a course that involves some complications, such as functional impairment, potential disfigurement or ulceration.^5^

In summary, vascular anomalies are a set of complex and heterogeneous pathological conditions, with regard to both their clinical presentation and their natural course. Because these lesions are usually located in visible areas, there are considerable chances that not only will systemic alterations appear, but also they will have great potential for psychosocial involvement in both the patient’s life and also the lives of the whole family. In fact, a multidisciplinary team is needed for treating these anomalies, and the treatment should be aimed towards better management of symptoms and complications, considering that the healing of these injuries is difficult and that resurgence of lesions occurs frequently. Hence, evidence concerning congenital vascular anomalies and hemangiomas is needed in order to improve the understanding of these diseases and the benefits of different types of treatment.

## OBJECTIVE

The aim of this review was to identify and summarize the evidence from Cochrane systematic reviews (SRs) regarding congenital vascular anomalies and hemangiomas, in order to establish better clinical decision-making.

## METHODS

### Design and setting

This was a review of Cochrane SRs conducted in the Division of Vascular and Endovascular Surgery, Universidade Federal de São Paulo, Brazil.

### Inclusion criteria

#### Types of participants

The participants included children and adults (both males and females) who had been diagnosed with congenital vascular anomalies or hemangiomas, without any restrictions regarding the site affected.

#### Types of interventions

We considered SRs that assessed any pharmacological intervention (e.g. beta-blocker agents) or non-pharmacological intervention (e.g. transdermal laser) for treating congenital vascular anomalies or hemangiomas. The focus of the studies included was to analyze different types of interventions for treating congenital vascular anomalies and hemangiomas, and the respective improvements. The main types of treatment referred to were pulsed-dye laser therapy, oral propranolol, oral prednisolone and conservative management.

#### Types of outcomes

We did not predefine the outcomes of interest. Rather, we considered all outcomes as reported in the SRs included.

#### Types of studies

All Cochrane SRs published thus far, about congenital vascular anomalies or hemangiomas, without restrictions regarding date of publication, were included. Withdrawn or outdated versions of SRs and protocols for SRs were considered not relevant.

#### Search for reviews

We conducted a systematic search in the Cochrane Database of Systematic Reviews on March 9, 2021. We used the following MeSH terms and related variants in the titles, abstracts and keywords: “Vascular malformations”, “Lymphatic abnormalities” and “Hemangioma”. The detailed search strategy is presented in Table 1.

**Table 1.** Search strategy and results from the Cochrane Database of Systematic Reviews

Lines	Search terms	Number of records
#1	MeSH descriptor: [Vascular Malformations] explode all trees	301
#2	MeSH descriptor: [Lymphatic Abnormalities] explode all trees	20
#3	(Lymphatic Abnormalit*) or (vascular malformation*)	597
#4	MeSH descriptor: [Hemangioma] explode all trees	171
#5	Hemangioma* or (Hemangioma* Intramuscular) or (Hemangioma* Histiocytoid) or Angioma or Chorioangioma* or Chorangioma*	473
#6	#1 #2 or #3 or #4 or #5	1041
#7	Cochrane reviews of intervention	156

#### Selection of reviews

Two researchers (HJGN and LCUN) independently evaluated the titles and abstracts to analyze whether the SRs fulfilled the inclusion criteria. Any disagreement was resolved by consulting other authors (DABK, RLGF, JCCBS and JEA). A total of three reviews fulfilled the inclusion criteria. The SRs were selected and summarized by two authors (HJGN and DABK) using previously developed forms to extract data from SRs, which had already been used in previous narrative reviews with this purpose.^5^ We extracted the following study characteristics:

- Participants: N randomized, N lost to follow-up/withdrawn, N analyzed, N of interest, mean age, age range, gender, condition of interest, inclusion criteria and exclusion criteria.
- Interventions: intervention and comparison characteristics, level of experience of the person carrying out the procedure, concomitant medications and medications excluded.
- Outcomes: primary and secondary outcomes specified and collected, and time points reported.
- Study methods: primary study design, number of primary studies and location, study setting and date of study.

#### Presentation of results

The results from the search and the SRs included were presented as a qualitative synthesis (descriptive approach).

### Ethics

No ethics committee approval was necessary since this was not a primary study and we did not deal directly with patients.

## RESULTS

### Search results

**Figure 1.**
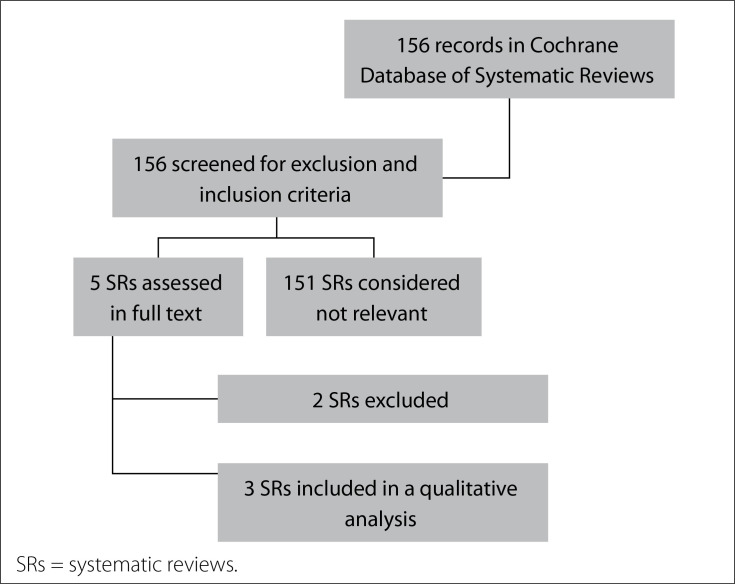
Study flow diagram. SRs = systematic reviews.

Our search strategy retrieved 156 references and, after screening the titles and abstracts, five SRs were preselected. After assessing the full texts, three reviews were found to fulfill the criteria for inclusion and were assessed in a qualitative synthesis (Figure 1).

### Reviews included

The latest versions of all the SRs included were published between 2011 and 2019.^13–15^ Details regarding the characteristics of interventions, comparisons, outcomes and certainty of evidence are presented in Table 2.

**Table 2.** Characteristics of interventions, comparisons, participants and main findings and the certainty of the evidence, as evaluated by means of the grading of recommendations, assessment, development and evaluation (GRADE) system

Review (primary studies, participants)	Interventions	Outcomes	Participants	Main findings	GRADE
Faurschou et al.^13^ (5 RCTs; 103 participants)	**Pulsed-dye laser and intense pulsed light**	Participant preference	Adults and children with port-wine stains	• Most patients preferred pulsed-dye laser over intense pulsed light	•N.A.
	**Pulsed-dye laser in association with cooling and pulsed dye laser alone**	Participant preference	Adults and children with port-wine stains	• Most patients preferred pulsed-dye laser in association with cooling, rather than pulsed-dye laser alone	•N.A.
	**Pulsed-dye laser and wait-and-see (active monitoring)**	Clearance	Children with port-wine stains	• There was no statistically relevant difference between these two approaches	•N.A.
		Skin atrophy		• 3.5 times more frequent with pulsed-dye laser	•N.A.
		Hypopigmentation		• 3.0 times more frequent with pulsed-dye laser	•N.A.
		Aesthetic appearance		• There was no statistically relevant difference between these two approaches	•N.A.
Novoa et al^14^ (28 RCTs; 1728 participants)	**Placebo and propranolol**	Clearance	Children with hemangiomas of the skin	• The likelihood of clearance after propranolol 1 mg/kg/day was 13.48 times greater than after placebo • The likelihood of clearance after propranolol 3 mg/kg/day was 16.6 times greater than after placebo	• Moderate
		Adverse events		• There was no difference between these two approaches	• Low
		Redness improvement, proportion of parents who considered that their children still had a problem, proportion of children who considered that they still had a problem, esthetic appearance and requirement for surgical correction		• There were no studies reporting this outcome	• N.A.
	**Topical timolol and placebo**	Clearance and subjective measurement of improvement	Children with hemangiomas of the skin	• There were no studies reporting this outcome	• N.A.
		Adverse events (bradycardia)		• There was no difference between these two approaches	• Low
		Volume reduction		• The likelihood of volume reduction after topical timolol maleate was 5.21 greater than with placebo	• Moderate
		Other measurements of resolution, as assessed by a clinician, at any follow-up: no redness		• The likelihood of no redness after topical timolol maleate was 8.11 times greater than with placebo	• Low
	**Topical bleomycin and placebo**	Shrinkage of lesions	Children with hemangiomas of the skin	• The shrinkage of lesions after bleomycin was 21 times greater than with placebo	•N.A.
	**Nd:YAG in association with oral propranolol versus Nd:YAG alone**	Clearance and superficial scars	Children with hemangiomas of the skin	• There was no clear difference between these two approaches	•N.A.
Novoa et al^14^ (28 RCTs; 1728 participants)	**Nd:YAG in association with oral propranolol versus oral propranolol alone**	Clearance	Children with hemangiomas of the skin	• The likelihood of clearance after Nd:YAG + oral propranolol was 8.44 times greater than with propranolol alone	•N.A.
		Superficial scars		• There was no clear difference between these two approaches	•N.A.
		Improvement ≥ 95%		• The likelihood was 2.83 times greater with Nd:YAG + oral propranolol than with oral propranolol alone	•N.A.
	**Propranolol and prednisolone**	Risk of complications	Children with hemangiomas of the skin	• The risk of complications after oral propranolol was 78% lower than after oral prednisolone	•N.A.
		Size reduction		• No clear difference was found	•N.A.
	**Oral propranolol + oral prednisolone versus oral propranolol alone**	Risk of adverse events	Children with hemangiomas of the skin	• The risk of adverse events after oral propranolol was 70% lower than with dual therapy	•N.A.
	**Oral propranolol + oral prednisolone versus oral prednisolone** **alone**	Adverse events	Children with hemangiomas of the skin	• No clear difference was found between these two types of treatment	•N.A.
		Size reduction		• There was no significant difference between dual therapy and oral prednisolone	•N.A.
	**Oral propranolol versus topical timolol**	Clearance and subjective measurement of improvement	Children with hemangiomas of the skin	• There were no studies reporting this outcome	• N.A.
		Adverse events (general adverse events)		• The risk was 7 times higher among participants randomized for propranolol	• Very low
		Other measurements of resolution, as assessed by a clinician, at any follow-up: no redness		• There was no statistically relevant difference between these two approaches	• Low
Zuurbier et al.^15^ (One RCT; 226 participants)	**Intervention and conservative management for treating brain arteriovenous malformations**	Risk of death or dependence	Adults with brain arteriovenous malformations	• 2.53 times greater for patients randomized for intervention	• Moderate
		Symptomatic intracranial hemorrhage		• The risk was 6.75 times higher for participants who underwent to intervention	• Moderate
		Epilepsy		• There was no statistically relevant difference between them	• Moderate

N.A. = not available; Nd:YAG = neodymium-doped yttrium aluminum garnet laser; RCTs = randomized controlled trials.

### Lasers or light sources for treating port-wine stains^13^

The aim of this SR was to study participant satisfaction with treatment of port-wine stains by means of laser and light sources, and the clinical efficacy and adverse events of this treatment. Five randomized clinical trials (RCTs) were identified, involving a total of 103 participants. The interventions and outcomes varied among the primary studies and therefore, could not be combined for numerical analysis.

#### Main findings

All of the primary studies described the participants’ level of satisfaction at less than six months after treatments with the pulsed-dye laser, intense pulsed light and Nd:YAG laser, and reported that the participants’ satisfaction was good or excellent, with regard to the degree of improvement attained.

Participant preference was analyzed in three of the five studies included, and most of the participants preferred pulsed-dye laser over intense pulsed light. The participants also preferred treatment with pulsed-dye laser in association with cooling, over treatment solely with pulsed-dye laser.

There was a significant clinical change of at least 20% in all the SRs regarding reduction of skin redness. All the studies determined the level of reduction in redness at one to three months after the final treatment. All five trials used the pulsed-dye laser, and, depending upon the setting, this resulted in more than 25% reduction in redness. The results reported were achieved after one to three sessions for up to six months postoperatively, in 50% to 100% of the participants. Adverse effects were considered in terms of their cosmetic aspect and were determined as either permanent or lasting longer than six months.

#### Complications

Few studies described short-term adverse effects occurring only in the first two weeks. Two primary studies reported that treatment with pulsed-dye laser alone was more painful than with pulsed-dye laser combined with cryogenic cooling. Three trials reported pigmentary complications in 3%-24% of the participants, such that the highest percentage occurred among Chinese participants with darker skin types. One case of scarring of the skin caused by high-dose laser was also reported. The trials included reported short-term side-effects such as pain, crusting and blistering in the first two weeks after the intervention.

#### Conclusion

Treatment of port-wine stains with pulsed-dye laser has clinical benefits, especially in relation to improvement of redness. However, it was not possible to compare the different types of treatments due to the small number of SRs involved in the studies and the absence of certainty regarding the evidence available, as determined through using validated tools like the ‘Grading of Recommendations, Assessment, Development and Evaluation’ (GRADE).

### Interventions for infantile hemangiomas of the skin^14^

This SR focused on assessing the effects of interventions for managing infantile hemangiomas in children. Twenty-eight primary studies were included, with a total of 1728 participants, in which 12 different kinds of interventions were analyzed. The most commonly used interventions were beta blockers, lasers, steroids, surgery and other types of treatment such as bleomycin and imiquimod. The primary outcomes analyzed were clearance (proportion of children with lesions completely cleared) and subjective measurements of improvement and adverse events secondary to each intervention over the short and long terms. The secondary outcomes were other measurements of resolution (i.e. surface area, lesion volume and lesion redness), the proportions of the parents and children who considered that the participant still had a problem, esthetic appearance and requirement for surgical correction. The quality of the evidence relating to the primary and secondary outcomes was assessed using the GRADE system.

#### Main findings and complications

Twenty-one studies used a two-arm design, six studies used a three-arm design and a single study used a four-arm, parallel group design. The numbers of children in the studies ranged from 12 to 460. Most of the studies had a greater number of females than males and the maximum age at enrollment at the beginning of the trial ranged from 14 weeks to five years. The median time taken for treatment was 24 weeks and the follow-up period ranged from seven days to 72 months.

The first comparison between pulsed-dye laser and the wait-and-see approach (active monitoring) included 143 children from two different trials. One study proved that there was no difference in terms of clearance, in comparing these two different approaches, with a risk ratio (RR) = 0.94 and 95% confidence interval (CI) = 0.62-1.42. Two different trials provided information about adverse events. In one of them, it was concluded that skin atrophy and hypopigmentation after pulsed-dye laser were more frequent, with RR = 3.46 (95% CI = 1.36-8.77) and RR = 3.05 (95% CI = 1.57-5.93), respectively. One study analyzed the proportion of parents who considered that their children still had a problem after treatment, during the follow-up period, and no clear difference was found in comparing pulsed-dye laser and the wait-and-see approach (RR = 1.24; 95% CI = 0.56-2.78). Regarding esthetic appearance after treatment, it was reported in one study that there was a better cosmetic outcome in seven children out of 11 after pulsed-dye laser therapy and in four out of 11 in the wait-and-see group, but that there was no statistically significant difference (RR = 1.75; 95% CI = 0.71-4.31).

The second comparison between placebo and propranolol treatments included information from three trials (312 children). One trial proved that the risk of clearance after administration of propranolol, 1 mg/kg/day, was 13.48 times greater than after placebo (RR = 13.48; 95% CI = 3.41-53.30). The likelihood of clearance after administration of propranolol, 3 mg/kg/day, was 16.6 times greater than after placebo (RR = 16.61; 95% CI = 4.22-65.34). In terms of adverse events, there was no significant difference between use of oral propranolol and placebo, at any doses. Also, there were no differences between these two different approaches, with regard to redness improvement, the proportion of parents who considered that their children still had a problem, the proportion of children who considered that they still had a problem, esthetic appearance or requirement for surgical correction.

Comparison of topical timolol and placebo treatments proved that there was no significant difference between them, with regard to clearance, subjective measurements of improvement or adverse events. One study demonstrated that volume reduction after use of topical timolol maleate was 5.21 times greater than after placebo (RR = 5.21; 95% CI = 1.28-21.21).

On the other hand, the analysis of topical bleomycin and placebo included one trial with 30 children. This trial suggested that most of the children treated with bleomycin reached clearance of lesions and shrinkage of lesions after use of bleomycin at a rate 21 times greater than through use of placebo (RR = 21.00; 95% CI = 1.34-328.86).

The analysis on neodymium-doped yttrium aluminum garnet (Nd:YAG) laser in association with oral propranolol versus Nd:YAG alone included two trials with a total of 107 children. The duration of treatment and follow-up was six months. There was no clear difference between these two types of treatment in terms of clearance and superficial scars. One of the studies proved that Nd:YAG laser + oral propranolol was 8.5 times more likely to show an improvement of at least 95%, compared with Nd:YAG laser alone.

On the other hand, comparison of Nd:YAG in association with oral propranolol versus oral propranolol alone proved that the likelihood of clearance after use of Nd:YAG laser + oral propranolol was 8.44 times greater than through use of propranolol alone (RR = 8.44; 95% CI = 1.14-62.66). There was no clear difference in terms of superficial scars (RR = 0.60; 95% CI = 0.05-7.63). Attainment of an improvement greater than or equal to 95% was 2.83 times more likely with Nd:YAG laser + oral propranolol than with oral propranolol alone (RR = 2.83; 95% CI = 1.42-5.67).

The comparison between propranolol and prednisolone included information from two trials, with a total of 39 children. These trials did not include any information regarding clearance and subjective measurement. One of the studies suggested that the risk of complications after use of oral propranolol was 78% lower than after use of oral prednisolone (RR = 0.22; 95% CI = 0.06-0.78). Neither of these studies found any clear differences in terms of size reduction, in comparing these two types of interventions.

The analyses on oral propranolol + oral prednisolone versus oral propranolol alone demonstrated that the risk of adverse events after use of oral propranolol was 70% lower than when dual therapy was used (RR = 0.30; 95% CI = 0.10-0.91; I^2^ = 0%), with no clear benefit regarding size reduction. However, the analysis on oral propranolol + oral prednisolone versus oral prednisolone alone demonstrated that there were no clear differences, in terms of adverse events, between these two types of treatment. Also, there was no significant difference between dual therapy and oral prednisolone, regarding size reduction.

#### Conclusion

Propranolol remains the standard treatment for infantile hemangiomas and is probably beneficial, in terms of clearance and reduction of hemangioma volume, compared with placebo.

### Interventions for treating brain arteriovenous malformations in adults^15^

The objective of this review was to determine the effectiveness and safety of different interventions, alone or in combination, for treating brain AVMs in adults, compared against each other, or with conservative management, in RCTs. The primary outcome was death or dependence due to any cause. The secondary outcomes included symptomatic intracranial hemorrhage, epilepsy, symptomatic radiation necrosis and quality of life. Only one study fulfilled the inclusion criteria for this review.

#### Main findings

The primary and secondary outcomes were available for 218 participants. During the first year, the relative risk of death or dependence for participants randomized to interventional treatment was 2.53 greater than for participants randomized to conservative management (RR = 2.53; 95% CI = 1.28-4.98). The total number of participants with symptomatic intracranial hemorrhage was also higher in the group with intervention (RR = 6.75; 95% CI = 2.07-21.96).

In terms of epilepsy, comparison between the study arm that underwent the intervention and the arm that was treated with conservative management demonstrated a RR of 1.14 (95% CI = 0.63-2.06).

#### Conclusion

Although the quality of evidence of this study was considered moderate, conservative management was superior to intervention with regard to functional outcome and symptomatic intracranial hemorrhage, over one year after randomization.

## DISCUSSION

Overall, there is a great variety of treatments for congenital vascular abnormalities and infantile hemangiomas and yet there is no consensus about which one is better.^5,6,8,11^ Each technique has its benefits and risks and the type of treatment should be based not only on the characteristics of the lesion, but also on the participant’s profile.

The first review described in this study suggested that treatment of port-wine stains with pulsed-dye laser improves the redness of these lesions. Pulsed-dye laser is considered to be the gold-standard treatment for port-wine stains,^16^ but the response to this treatment varies according to the patient’s age, lesion location, the frequency used and the intervals between sessions.^17^ Some studies have suggested that port-wine stains located proximally to the limbs tend to have better results than those that are distal to the limbs, from treatment with pulsed-dye laser.^18^ The SR described above included a small number of studies and, therefore, it was not possible to properly analyze these factors or compare different types of treatment.

The second SR compared a number of types of treatment and suggested that propranolol remains the standard treatment for infantile hemangiomas and is probably beneficial in terms of clearance and reduction of hemangioma volume. Although this review suggested that there were no significant differences in terms of improvement and adverse events, in comparing the use of propranolol at 1 mg/kg/day and 3 mg/kg/day with use of placebo, some reports in the literature have suggested that there is higher incidence of adverse events related to propranolol when it is administered at higher doses.^19^ Perhaps the number of participants included was not enough to compare the effects of propranolol at different doses.

Regarding treatment of brain AVMs, our study suggested that conservative management was superior to intervention. However, there is no consensus about this. There is evidence from different studies suggesting that conservative management may be associated with worse outcomes.^20,21^

The major limitation of this review was the small number of SRs included. There were also the facts that a great variety of treatments were presented and different comparisons were made between small numbers of participants. The results may have been influenced by the ages of the participants, locations of lesions and individual characteristics of each participant. These matters were not stratified in some of the analyses.

Nonetheless, our study had intrinsic value with regard to providing information about different types of treatment and their benefits and complications, especially considering the small number of SRs published thus far in the literature. This may help physicians to improve clinical care and medical treatment.

## CONCLUSION

Despite the controversies in the literature regarding the treatment of congenital vascular abnormalities and hemangiomas, Cochrane SRs suggest that treatment of port-wine stains with pulsed-dye laser improves redness; propranolol remains the best option for infantile hemangiomas; and conservative management seems to be superior to surgical intervention for treating brain AVMs.

Additional evidence is needed to better understand the different types of treatments and their benefits and complications, along with the clinical results after a long period of follow-up.
